# Case of Spontaneous Evolution of the Fœtus

**Published:** 1824-09

**Authors:** Robert Brown

**Affiliations:** Preston; Fellow of the Royal College of Surgeons, &c. London.


### Art. V.—*Case of Spontaneous Evolution of the Fœtus.* By Robert Brown, Esq. Preston; Fellow of the Royal College of Surgeons, &c. London.

Mrs. Thomas T., ætatis twenty-six, of a constitution moderately strong, the mother of three children, between seven and eight months advanced in pregnancy; on Saturday evening, June 26th, was severely shaken whilst returning in a coach from a visit at a distance, and also very much distressed in her mind from some dometic cause. On the following day, she experienced trifling pains in the belly and loins, which continued progressively to increase. I was called to her at half-past six o'clock this morning (June 28, 1824). The pains were strong and urgent. I directed the patient immediately to lie down in the usual position on the bed. On examination per vaginam, I found the membranes very full and tense, and acting strongly against the os externum. During the second pain, I accidentally and unintentionally ruptured them; when a copious discharge of liquor amnii took place. The pains from this time became very trifling and long distant; and the discharge became hemorrhagic, and continued to be poured off in large quantity, both during and in the absence of pain, for about an hour and a half. On extending my search for the cause of the sanguineous evacuation, I found the placenta (as I previously conjectured) separated from its original place of attachment, occupying one edge of the sacral portion of the os uteri, at the superior aperture of the pelvis; and which I could only ascertain and arrive at by first breaking through the large clots of coagulated blood, which filled up the hollow of the sacrum, &c.

After many fruitless attempts to discover the presenting part of the child, I at length felt the right hand of the fœtus in the middle of the right ileo-pectineal margin. The os uteri was only so much dilated as to admit my hand to the knuckle, upon which it acted most painfully; the patient complaining sometimes of great pain in the loins, but of still greater in the abdomen. In the interval of the pains, I endeavoured to pass my hand, so as to bring down one or both feet of the fœtus; but, after giving the patient about three ordinary-sized tea-spoonfuls of tinct. opii, at twice, and four hours' deliberate and persevering attempt, I could never reach further than the ileum of the child, such was the spasmodic action which the uterus manifested. At times, however, during my endeavours to turn the child, a surprising degree of dilatation took place at the cervix and lower part of the uterus, whilst the upper firmly encircled the body of the fœtus, rendering all attempts to reach the feet, for some time at least, both futile and dangerous.

Having employed all the tinct. opii which I brought with me, and fearing to do mischief by continuing the attempt to deliver my patient under the existing circumstances, I set off home for more; but, on the way, called upon my friend, Dr. Moore, to borrow some, and invited him to accompany me. As we passed out at his door, a messenger came running to send me back. When I arrived, an attendant was officiating. The breech and half of the body of a dead female child were protruding at the os externum; and a pain soon following, the whole was expelled, preceded (as we were informed) by a large mass of coagulated blood. Immediately on separating the child, the placenta was found low down in the vagina, and was speedily, and with the greatest ease, removed.

Evolution of the fœtus was in this case a most fortunate, but a very unexpected and agreeable, issue: the extremely violent and long-continued spasmodic action of the uterus rendered the effort to turn both unavailing and perilous.

On withdrawing my hand from the uterine cavity, the patient appeared disposed to sleep, arising, probably, from the quantity of laudanum given; which also was, no doubt, favourable to the change which so soon followed, as she only suffered from three or four pains from its taking place to the expulsion of the child. The child was of the size usual at this period of utero-gestation, and was alive when I made the attempt to turn. The hand and arm that presented were greatly discoloured, from the unavoidable pressure made upon the upper part of the arm against the brim of the pelvis, in the attempt at the operation of turning.

The recovery of the patient has been uninterruptedly good.

The following observations on spontaneous evolution of the fœtus, are taken from the last edition of a very valuable and highly useful practical work, the "Synopsis of the various Kinds of difficult Parturition," the production of a zealous and experienced practitioner, Dr. Merriman: they coincide so exactly with the remarks which I had intended to have made on this occasion, that I have preferred quoting them; being, in my opinion, exceedingly applicable, and more likely to be considered orthodox, than any thing which I could have furnished.

"The occurrence of the spontaneous evolution has, however, been comparatively so rare, that no man would be justifiable in simply relying upon it. The knowledge that it has sometimes happened, may, indeed, under some circumstances of extreme resistance to the passage of the hand into the uterus, reconcile us to the delay which I have recommended; but we should never allow it to operate upon our minds, so as to induce us to neglect the proper means and proper time of turning, when we have it in our power. It is the duty of the accoucheur, on all occasions, to give to nature every possible opportunity of exerting herself for the relief of the patient; but it is equally his duty, when nature becomes embarrassed and oppressed, to interpose the timely assistance of art, lest nature, being compelled to relinquish the task, the patient shall fall a sacrifice to the delay."

*Preston, Lancashire; August,* 1824.

